# A Pilot Study of FDG-PET/CT in Polycythemia Vera Using Global Analysis Techniques

**DOI:** 10.22038/aojnmb.2019.41192.1278

**Published:** 2020

**Authors:** Cyrus Ayubcha, Hitomi Hosoya, Siavash Mehdizadeh Seraj, Mahdi Zirakchian Zadeh, Thomas Werner M.S.E, Abass Alavi

**Affiliations:** 1 Department of Radiology, Perelman School of Medicine, University of Pennsylvania, PA, United States; 2 Department of Medicine, Pennsylvania Hospital, University of Pennsylvania, Philadelphia, PA, United States

**Keywords:** Polycythemia Vera, FDG-PET/CT, Myeloproliferative Neoplasm, Non-Invasive Procedure, Global Analysis

## Abstract

**Objective(s):**

Functional imaging presents a non-invasive process that may capture the hyper-metabolic nature of red bone marrow in myeloproliferative neoplasms, such as polycythemia vera (PV).

**Methods:**

This study analyzed the FDG-PET/CT scans (n=12) of six patients diagnosed with PV and six age-sex matched controls using a quantitative global analysis methodology.

**Results:**

All PV patients had elevated activities in the bone marrow of each skeletal structure as compared to matched controls with respect to mean standardized uptake value (femoral neck p=0.01, lumbar spine p=0.02, pelvis p=0.002, sternum p=0.04). Notable variations in splenic uptake were observed among the treated and untreated PV patients.

**Conclusion:**

Our study exemplifies the potential utility of PET in reflecting hyperactive bone marrow activity related to PV. Future studies may further substantiate and elaborate on the use of PET-derived metabolic data in PV.

## Introduction

 Polycythemia vera (PV) is a myeloproliferative neoplasm characterized by abnormal proliferation of hematopoietic stem cells, which can lead to life-threatening thrombotic events (1). Diagnosis is made based on hemoglobin/hematocrit levels, bone marrow biopsy (BMBx) findings, and presence of JAK2 mutations per the WHO criteria (1). The treatment for PV varies according to an individual’s prospective risk of recurrent thrombosis. Treatments include low-dose aspirin and phlebotomies in low-risk patients, while hydroxyurea (HU) or ruxolitinib is available for high-risk patients (2). 

The set of diagnosis criteria utilizes BMBx as a major criterion (1). Unfortunately, BMBx is an invasive procedure, which can only represent a local pathology. Alternatively, radio-nucleotide imaging techniques have potential to provide a comprehensive understanding of myeloproliferative neoplasm via a non-invasive assessment of cellular glycolysis rates as demonstrated by the uptake of radioactive glucose analogues such as ^18^F-fluorodeoxyglucose (FDG) (3). Accordingly, radio- nucleotide imaging could potentially monitor treatment response in hematological disorders that involve hyper-active bone marrow such as polycythemia vera. While previous studies have imaged bone marrow disorders (3) and case studies of PV patients with ^18^F-fluorodeoxyglucose-positron emission tomography/computed tomography (FDG-PET/CT) (4-6) have qualitatively assessed individual scans, this report quantitatively assesses activities among a group of control-matched subjects and juxtaposes clinical data in such context. As such, we explore the potential scientific and clinical relevance of FDG-PET/CT in the assessing PV. 

## Methods

***Subjects:***

 After this study was approved by the institutional review board and all subjects provided informed consent, we performed a retrospective study. Using the database of FDG-PET/CT scans from the Hospital of the University of Pennsylvania, all patients diagnosed with PV in their medical records were selected. This initial cohort of PV patients was further refined as patient records were reviewed to include only patient scans (n=6) obtained as part of the initial staging of malignancies, prior to initiation of any chemotherapy or immunomodulatory agents that some patients received after the scan. Control group patients (n=6) were selected to match the sex and age (±3 years) of the respective diseased subjects. All control subjects were selected from a larger group of subjects, all of whom were determined to possess benign solitary pulmonary nodules via an FDG-PET/CT scan. The patient records of all subjects in the study were further reviewed to exclude potentially confounding diseases or treatments that could unduly alter bone marrow activity, namely diagnosed cardiovascular disease, diabetes, immune disorders, hematological malignancies, anemia, and erythropoietin therapy. Reading of the scans by a qualified radiologist further excluded subjects presenting metastatic involvement of the skeleton or other musculoskeletal abnormalities such as fractures and degenerative conditions of the spine in all individuals. Inter-group and intra-group scanning procedures remained uniform among all scans. Each individual pair was matched for approximately similar ratios of ^18^F-FDG injection dosage and time from intravenously injection to scan (mean =62±4 minutes). All PET/CT scans were performed from the base of the skull to the mid-thigh.

***Segmentations and Analysis:***

**Figure 1:**
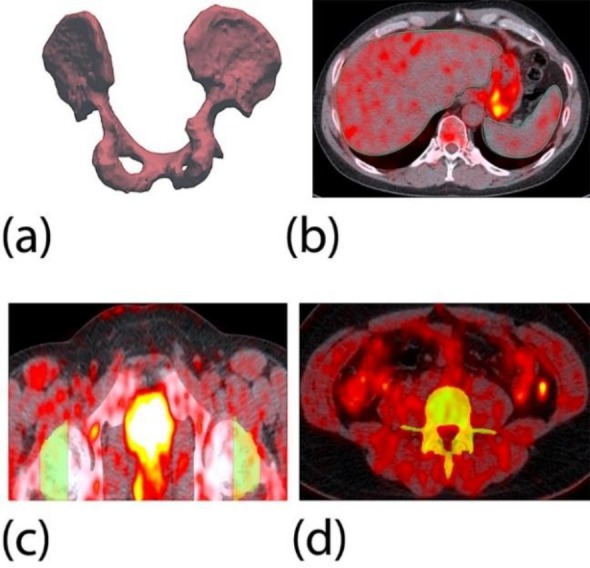
The set of images illustrates the various segmentation methodologies. (a) A three-dimensional volumetric representation of an exemplary pelvis ROI. (b) A manually applied ROI to the liver (green) and spleen (blue). (c) An axial slice of a femoral neck ROI as applied by a semi-automated technique. (d) An axial slice of a spine ROI as applied by a semi-automated technique where the spinal cord is excluded

 The CT and PET data were coregistered using OsiriX software; Pixmeo SARL Bernex, Switzerland (version 7.04). A semi-automated three-dimensional segmentation algorithm was applied based on Hounsfield unit thresholds. Each scan's three-dimensional maximum intensity projection, as defined by the CT, was used to isolate the anatomical boundaries of relevant structures. The lumbar spine was defined from the L1 to L5 vertebrae. The femoral neck's medial and lateral boundaries were defined from epiphyseal line to the intertrochanteric ridge. The pelvis analysis included the whole pelvis after the sacrum was removed. Various particular threshold ranges were specifically applied to each skeletal structure (e.g. femoral neck, lumbar spine, pelvis, sternum). The encapsulating cortical tissues of each structure were included in the initial automated region of interest (ROI) so that a closing procedure was applied to comprehensively include the complete skeletal structure as a global ROI. Soft tissue structures (e.g. liver, spleen) were easily discernable on the CT imaging so that a global ROI was derived from manually applied regions through an axial progression of slices (Figure 1).

 Subsequent PET data was exported from the ROIs to determine the average uptake, maximum pixel of uptake and area of each axial ROI. A global SUV_mean_ was derived based on previously published methods (7, 8), where the total uptake in the global ROI was divided by the total structural volume. Given contamination concerns, the Grubb’s Outlier Test was applied to the data set of maximum pixel uptake values per each axial slice of the global ROI; the remaining maximum pixel of uptake was then used as the global SUV_max_. The global SUV_mean_ and SUV_max_ were augmented based on the Janmahasatian equation (9) to better standardize the values for variations in lean body mass (lbm) composition for the subjects. Finally, a SUV_mean-lbm_ and SUV_max-lbm_ was derived for each diseased and matched control subject. 

## Results

 A qualified hematologist retrospectively assessed the PV diagnosis of six PV patients. Out of six, two had documented JAK2 mutation, one had wild type, and three did not have documentation of JAK2 status. When the patients underwent FDG-PET/CT scan, three patients were taking hydroxyurea (HU), three had recently received phlebotomies (PHL) of which one was also taking aspirin (ASA) (Table 1).

**Table 1:** Uptake of Respective Populations

Group Characteristics	Structure	SUV_mean-lbm_			SUV_max-lbm_		
		Mean Patient	Mean Control	p-value	MeanPatient	Mean Control	p-value
Complete Patient Group (n=6)	Femoral Neck	0.71±0.09	0.53±0.15	0.01*	1.41±0.31	1.16±0.24	insignificant
Complete Patient Group (n=6)	Lumbar Spine	1.13±0.24	0.88±0.22	0.02*	2.45±1.11	1.63±0.50	insignificant
Complete Patient Group (n=6)	Pelvis	0.80±0.08	0.63±0.09	0.002*	2.39±0.65	1.87±0.37	insignificant
Complete Patient Group (n=6)	Sternum	1.06±0.36	0.72±0.14	0.04*	1.66±0.67	1.09±0.21	0.004*
Untreated Patient Subgroup (n=3)	Femoral Neck	0.71±0.13	0.52±0.22	N/A	1.59±0.46	1.13±0.32	N/A
Untreated Patient Subgroup (n=3)	Lumbar Spine	1.84±0.18	0.93±0.31	N/A	3.41±0.95	1.87±0.75	N/A
Untreated Patient Subgroup (n=3)	Pelvis	0.85±0.11	0.66±0.09	N/A	2.92±0.32	1.99±0.39	N/A
Untreated Patient Subgroup (n=3)	Sternum	1.22±0.38	0.71±0.17	N/A	1.98±0.92	1.12±0.29	N/A
Untreated Patient Subgroup (n=3)	Spleen	1.45±0.19	1.16±0.23	N/A	2.53±0.60	1.67±0.32	N/A
Treated Patient Subgroup (n=3)	Femoral Neck	0.75 ± 0.04	0.51 ± 0.07	N/A	1.28 ± 0.08	1.22±0.25	N/A
Treated Patient Subgroup (n=3)	Lumbar Spine	1.02±0.17	0.76±0.03	N/A	1.79±0.58	1.41±0.21	N/A
Treated Patient Subgroup (n=3)	Pelvis	0.76±0.02	0.63±0.11	N/A	1.77±0.32	1.77±0.46	N/A
Treated Patient Subgroup (n=3)	Sternum	0.99±0.43	0.77±0.12	N/A	1.56±0.34	1.11±0.18	N/A
Treated Patient Subgroup (n=3)	Spleen	0.92±0.13	1.18±0.11	N/A	1.16±0.14	1.53±0.43	N/A

Table 1 **.** The table notes the mean±standard deviation of SUV_mean-lbm_ and SUV_max-lbm_ for both the PV and control populations. The p-values for the Mann-Whitney U test were also presented to indicate whether the elevated uptake values of the PV population were statistically distinct from the control population. Statistical tests were not performed upon the treated and untreated subgroups, as sample size was a concern. Liver uptake was unremarkable among all groups and subgroups

**Table 2:** Percentage Change in Uptake between Populations

Patient	PV Determination (Clinical History)	Treatment	Structural FDG Uptake: Percentage Change from Control: SUVmean-lbm, SUVmax-lbm					
			Femoral Neck	Lumbar Spine	Pelvis	Sternum	Spleen	Liver
1	Unclear (Diagnosed, JAK2 Unavailable)	Phlebotomy	+86% +39%	+119% +157%	+30% +56%	+75% +62%	+71% +56%	+96% -23%
2	Likely Secondary (Smoker, JAK2-)	Aspirin, Phlebotomy	+40% +39%	+27% +74%	+34% +59%	+100% +24%	+11% +15%	-34% -40%
3	Confirmed (JAK2+)	Phlebotomy	+11% +40%	+17% +51%	+22% +71%	+42% +35%	+7% +88%	+6% -8%
4	Confirmed (JAK2+)	Hydroxyurea	+66% +0%	+13% +10%	+52% +1%	+80% +58%	-30% -15%	-42% -37%
5	Likely Primary (JAK2 Unavaliable)	Hydroxyurea	+27% +27%	+31% +13%	+12% -8%	+66% +65%	-33% -30%	-12% -1%
6	Likely Primary (JAK2 Unavaliable)	Hydroxyurea	+54% -5%	+48% +52%	+8% +11%	+40% +5%	-2% -30%	+9% -42%

Table 2 **.** The table presents patient clinical data along with percentage change between each diseased and control matched pairs per SUV_mean-lbm_ and SUV_max-lbm_ within each relevant anatomical structure

## Discussion

 The enhanced metabolic activity within the skeletal structures of PV patients was visually observable not only with respect to magnitude of FDG uptake but also in the expanded dispersion of activity throughout the bones as compared to normal patients. In this study, we further move to the quantitative assessment of individual scans among the control-matched subject pairings in order to explore a potential objective clinical relevance of FDG-PET/CT in the diagnosis and assessment of PV. Our study found, that the quantitative uptake values coincided remarkably with the clinical trends unique to each patient. For example, patients treated with phlebotomy alone expressed the most elevated marrow activities, while those treated with HU conferred less elevated activities, which may reflect the effectiveness of HU in depressing marrow activity (10). 

**Figure 2:**
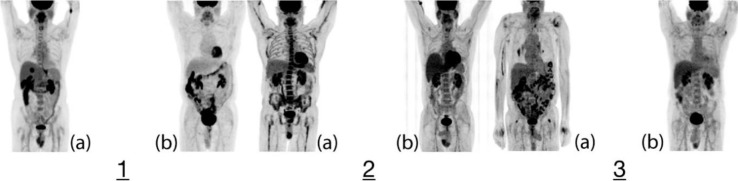
The following set of images show the whole-body FDG uptake of each set of case-control matched pairing where the respective PV patients had not received treatments. (1a) Patient 1’s clinical history of over-productive marrow, as evidenced by history of phlebotomies, aligns with the elevated, dispersed marrow activity presented by the FDG uptake. The qualitative FDG uptake of the PV patient’s marrow as compared to the (1b) control subject can be seen as elevated; such observable, elevated uptake aligns with quantitative values of bone marrow activity, namely in the spine, pelvis and femoral neck. Otherwise, the diseased patient was noted to confer an avid hepatic lesion and moderate uptake in the esophageal wall, both suspicious for malignancy, though the latter may involve an inflammatory etiology. (2a) Patient 2 lacked the JAK2 mutation and likely has secondary polycythemia. The diffuse heterogeneous FDG uptake is notably observed in the patient’s axial and appendicular skeleton as opposed to the (2b) control subject. Quantitative parameters of FDG uptake were elevated to further evidence hyper-metabolic bone marrow in the PV patient. The PV patient also was found to have focal FDG uptake at the GE junction, which aligns with a biopsy-proven adenocarcinoma. Focal uptake within the mid posterior mediastinum was suspicious for metastasis within a subcarinal lymph node. (3a) Patient 3 was confirmed to have the JAK2 mutation; however, the PET scans make it difficult to visually discern the increased skeletal uptake as compared to the (3b) matched control; elevated uptake is better captured in our quantitative analysis. Unrelatedly, the scan found multiple hypermetabolic foci of FDG uptake in the thyroid gland and lungs suspicious of potential malignancies

**Figure 3:**
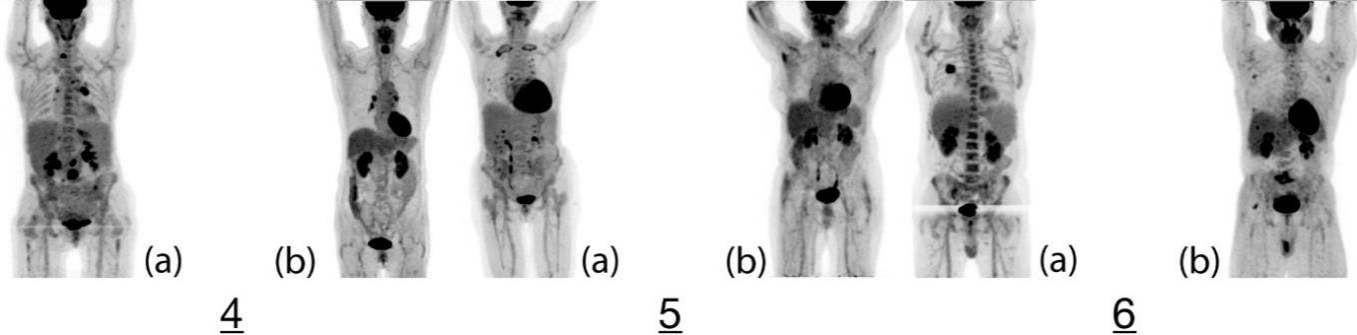
The following set of images show the whole-body FDG uptake of each set of case-control matched pairing where the respective PV patients had received treatments. (4a) Patient 4 was JAK2 positive but was also taking HU during the period of the PET scan. Visual inspection may indicate slightly elevated FDG uptake in the skeletal structures as compared to the (4b) control subject, which is confirmed by the moderate degree of deviance in the qualitative analysis. In respect to previous pairings, the PET data may indicate some degree of response to treatment in managing PV; though the remaining elevated activity may still relate to active bone marrow. Otherwise, the diseased subject harbors a necrotic hypermetabolic mass in the duodenum and an FDG-avid pulmonary nodule. (5a) Patient 5 was also taking HU over the period of time in which the scan was performed. The quantitatively minimal degree of bone marrow FDG uptake deviance between the (5b) control and patient may indicate a partial response to HU. However, increased localized uptake along the left mediastinum and hilum without may represent sites of extramedullary hematopoiesis related to underlying PV. This patient was not found to confer any nefarious lesions. (6a) Patient 6 was taking HU over the period of time in which the scan was performed. There was a visually observable increase in FDG activity in the patient as compared to the (6b) control subject throughout the skeleton as both observed qualitatively and measured quantitatively. The less extreme quantitative values may be related to a response to HU. Otherwise, a hypermetabolic right lung mass was noted with potential, though unlikely, metastatic involvement of the AP window, subcarinal lymph nodes and right adrenal gland

 Further, visual inspection aligned with quantitative parameters to indicate elevated FDG uptake within the skeletal structures of PV patients as compared to the control subjects. The results not only align with previous studies involving increased FDG uptake in bone marrow in myeloproliferative conditions (3) but also previous FDG-PET/CT case studies in PV, which qualitatively reflect hyper-functioning bone marrow related to PV (4-6). To this point, no other study has examined PV with respect to FDG-PET/CT beyond isolated case studies or otherwise involved controls subjects, treated patients or quantitative analysis. Our study also found that subjects treated with HU conferred lower splenic activities than controls. Though it is difficult to explain such depressed activity in these treated PV patients, the pharmaceutical action of HU (10) may be related. Otherwise, the increased splenic activity in the other untreated PV patients may be likely related to PV. The liver values for all patients were generally unremarkable. To note, the presented results seem to support the utility of SUV_mean_ as opposed to SUV_max _for such a global analysis methodology (11) (Figure 2, 3). 

 It is to note that while controls were "normal" by clinical and radiological means, there are certain random variations that may have influenced the control subjects to express activities slightly above or below the expected SUV. Also, the retrospective nature of this study and the limited number of subjects, as dictated by the rarity of the disease and coincidental application of PET, limits the scope of a definitive causative conclusion. Still, this study’s preliminary data does positively support the application of radioactive glucose analogues in quantifying marrow activities related to PV. Based upon our findings, a prospective study to assess a larger population prior and during treatment may establish FDG-PET/CT as potential diagnosis criteria for PV in replacement of invasive and perilous (12) procedures like BMBx with non-invasive procedures like PET. 

## Conclusion

 Our study indicates a potential utility of PET in reflecting the elevated glycolysis rates in vivo of hyperactive bone marrows and treatment responses related to PV. Accordingly, we lay the basis for future studies to justify the expanded use of PET in PV.

## Conflict of Interest

 The authors declare no conflict of interest. 

## Ethical Approval

 All procedures performed in studies involving human participants were in accordance with the ethical standards of the institutional, national research committee and with the 1964 Helsinki declaration and its later amendments or comparable ethical standards. 

## Informed Consent

 Informed consent was obtained from all individual participants included in the study.
